# Successful Transabdominal Cerclage for Recurrent Second‐Trimester Loss in a Low‐Resource Environment: A Case Report

**DOI:** 10.1155/crog/5373643

**Published:** 2026-08-12

**Authors:** Rex Mawuli Kwadjo Djokoto, Edward Anabila Agana, Kingsley Afreh Nduroh, Johnny Arthur-Komeh, Opei Adarkwa, Wilfred Kwamina Sam-Awortwi, Kwabena Addow Opare-Addo, Andrew Panyin Vormawor, Anthony Amanfo Ofori

**Affiliations:** ^1^ Department of Obstetrics and Gynaecology, Kwame Nkrumah University of Science and Technology, Kumasi, Ghana, knust.edu.gh; ^2^ Department of Obstetrics and Gynaecology, Oak Specialist Hospital, Kumasi, Ghana, unicamp.br; ^3^ Department of Obstetrics and Gynaecology, Komfo Anokye Teaching Hospital, Kumasi, Ghana, kathhsp.org; ^4^ Department of Obstetrics and Gynaecology, Powerhouse Hospital, Kumasi, Ghana, unicamp.br; ^5^ Department of Obstetrics and Gynaecology, Manhyia Government Hospital, Kumasi, Ghana, unicamp.br; ^6^ Department of Anaesthesiology and Intensive Care, Kwame Nkrumah University of Science and Technology, Kumasi, Ghana, knust.edu.gh

**Keywords:** case report, cervical insufficiency, Ghana, obstetrics, recurrent pregnancy loss, transabdominal cerclage

## Abstract

Cervical insufficiency is a significant cause of recurrent midtrimester pregnancy loss. Although transabdominal cerclage (TAC) is an established option for selected patients with severe cervical insufficiency, its application and outcomes remain undocumented in West African clinical settings. This case report documents the first use of TAC in Ghana. Three Ghanaian women, aged 32–33, with severe cervical insufficiency and a cumulative history of eight prior midtrimester losses and multiple failed transvaginal cerclages, underwent open TAC using 5‐mm Mersilene tape between 12 and 13 weeks of gestation at Oak Specialist Hospital in Kumasi, Ghana. All three reported pregnancies resulted in live births, yielding five neonates. Deliveries occurred at 38 weeks 4 days (singleton), 35 weeks 2 days (singleton) and 30 weeks 1 day (triplet gestation complicated by preeclampsia and peripartum cardiomyopathy). No major intraoperative or postoperative complications directly attributable to the surgery were observed. This case report demonstrates that TAC is a feasible intervention for severe cervical insufficiency in a Ghanaian specialist hospital setting. The preliminary outcomes are encouraging, but larger studies are needed to establish efficacy and generalizability across the West African region.

## 1. Background

Cervical insufficiency, defined as painless dilatation and effacement of the uterine cervix during the second trimester, represents a formidable challenge in modern obstetrics [1]. It is a primary cause of recurrent midtrimester pregnancy loss and extreme preterm birth, affecting approximately 1%–2% of all pregnancies and implicated in up to 15% of recurrent second‐trimester losses [2]. The underlying pathophysiology involves a loss of the cervical stroma′s structural and biomechanical integrity, which is commonly acquired through iatrogenic trauma, such as mechanical dilation or cone biopsy [3].

The conventional intervention, transvaginal cervical cerclage (TVC), has variable efficacy, with fetal survival rates typically ranging from 70% to 85%. Furthermore, TVC is often deemed technically difficult or impossible in patients presenting with a severely scarred or significantly shortened cervix [4–6].

Transabdominal cerclage (TAC), first described in 1965 by Benson and Durfee [7], overcomes these limitations by placing a permanent suture high at the cervico‐isthmic junction, offering superior biomechanical support. Global evidence consistently reports live birth rates of 85%–95% with TAC, positioning it as an established option for selected high‐risk patients who have experienced prior TVC failures or who possess prohibitive cervical anatomy [8].

Lotgering et al. [8] reported a landmark series of 101 pregnancies after TAC, documenting 93.5% neonatal survival compared with 27.5% before cerclage. Ades et al. [9] demonstrated 98.5% perinatal survival across 121 pregnancies following laparoscopic TAC, establishing the modern benchmark for the procedure. A recent meta‐analysis by Hulshoff et al. [10], encompassing 83 studies and 3398 patients, confirmed that both open and laparoscopic TAC approaches yield comparable perinatal outcomes, with survival rates exceeding 90%.

Despite its documented success worldwide, a profound knowledge and practice gap persists in sub‐Saharan Africa concerning TAC implementation and outcomes. A search of major medical databases reveals a complete absence of published data on this procedure from West Africa. This study is aimed at bridging this gap by documenting the first cases of TAC performed in Ghana, thereby establishing local feasibility and clinical outcomes.

## 2. Case Presentation

We present a detailed series of three cases involving women who underwent TAC at Oak Specialist Hospital, a private tertiary facility in Kumasi, Ghana, between January 2022 and July 2025. All participants provided informed consent for the use of their anonymized data.

Table 1 summarises the demographic, clinical and outcome data for the case series.

**Table 1:** Demographic, clinical and outcome data for the case report.

Parameter	Case 1	Case 2	Case 3
Age (years)	32	32	33
Ethnicity	Akan	Fanti	Akan
Occupation	Nurse	Banker	Self‐employed
Gravidity/parity	G5P1+3	G5P0+4	G4P0+3
Prior midtrimester losses	3	3	3
Prior failed TVC	2	2	2
Other pregnancy history	—	1 Ectopic pregnancy	—
Preoperative cervical length (mm)	15	10	8
Gestational age at TAC (weeks)	13+3	12+5	12+5
Operative time (min)	75	140	80
Estimated blood loss (mL)	50	200	70
Gestational age at delivery (weeks)	38+4	30+1	35+2
Mode of delivery	Elective CS	Emergency CS	Emergency CS
Birth weight (g)	3200	1100; 1050; 950	2450
Neonatal outcome	Healthy singleton	3 Live neonates (NICU)	Healthy singleton
Maternal complications	None	Preeclampsia, PPCM	None

### 2.1. Surgical Technique (All Three Cases)

After spinal anaesthesia, a low transverse abdominal incision was made. The vesicouterine peritoneum was incised and the bladder was mobilised inferiorly to expose the cervico‐isthmic junction. Bilateral windows were created in the broad ligament medial to the uterine vessels. A 5‐mm Mersilene tape was passed through each window at the level of the cervico‐isthmic junction, just medial to the uterine vessels and at the junction of the cervix and lower uterine segment. The tape was tied anteriorly, with the knot positioned on the anterior surface of the cervico‐isthmic junction. Haemostasis was confirmed, and the bladder peritoneum was reapproximated. All three patients received vaginal progesterone (400 mg daily) postoperatively until 36 weeks of gestation for 2 and till 30 weeks for the triplet mother. The 12–13‐week gestational timing was selected because it follows first‐trimester viability confirmation and nuchal screening while remaining early enough to precede the typical window of cervical insufficiency manifestation (14–24 weeks).

#### 2.1.1. Case 1

A 32‐year‐old Akan nurse (G5P1+3) presented with a history of recurrent pregnancy loss: three prior midtrimester losses and two previously failed transvaginal cerclages. Preoperative transvaginal cervical length was 15 mm. She underwent open laparotomy TAC at 13 weeks 3 days of gestation with a 5‐mm Mersilene tape placed at the cervico‐isthmic junction. Operative time was 75 min, with an estimated blood loss (EBL) of 50 mL. The postoperative course was uncomplicated. The pregnancy reached term, resulting in delivery of a healthy singleton infant via elective caesarean section at 38 weeks 4 days, birth weight 3200 g.

#### 2.1.2. Case 2

A 32‐year‐old Fanti banker (G5P0+4) presented with a history including three previous second‐trimester losses and one ectopic pregnancy. She had undergone two failed transvaginal cerclages. Preoperative cervical length was 10 mm. She underwent open TAC at 12 weeks 5 days of gestation. Operative time was 140 min, with an EBL of 200 mL. This pregnancy resulted in a triplet gestation, which was complicated in the third trimester by preeclampsia and peripartum cardiomyopathy, necessitating emergency caesarean section at 30 weeks 1 day. She delivered three live neonates (birth weights 1100, 1050, and 950 g), all of whom required NICU admission for prematurity.

Note that Case 2 involves a high‐order multiple gestation (triplets), which is a major confounder. The contributions of multiple‐gestation‐related risks (preeclampsia, peripartum cardiomyopathy and preterm delivery) cannot be disentangled from the effect of cerclage. This case is therefore discussed separately and should not be pooled with the singleton cases for efficacy assessment.

#### 2.1.3. Case 3

A 33‐year‐old Akan self‐employed woman (G4P0+3) was managed due to three previous second‐trimester losses and two failed transvaginal cerclages. Preoperative cervical length was 8 mm. The procedure was performed via open laparotomy at 12 weeks 5 days of gestation. Operative time was 80 min, with an EBL of 70 mL. Recovery was uneventful. The pregnancy was complicated by preterm labour, leading to emergency caesarean section at 35 weeks 2 days. She delivered a healthy singleton infant weighing 2450 g.

### 2.2. Adjunctive Therapy

All three patients received vaginal progesterone 400 mg daily from the day of surgery until 36 weeks of gestation and 30 weeks for the triplet mother, consistent with standard postoperative protocols for cervical cerclage.

## 3. Discussion

This case report presents the first documented clinical evidence regarding the use of TAC for severe cervical insufficiency in Ghana and the wider West African subregion. The primary finding is that TAC is a feasible intervention within a Ghanaian specialist hospital setting. In a cohort of three women with a cumulative history of eight prior second‐trimester losses and multiple failed TVCs, all three reported pregnancies resulted in live births, yielding five neonates. This outcome is consistent with published international benchmarks, where fetal survival rates following TAC exceed 90% [10].

The perinatal outcomes in this series are presented separately for singletons and the triplet gestation. For the two singleton cases, delivery at term (38 weeks 4 days, Case 1) and late preterm (35 weeks 2 days, Case 3) are consistent with the mean gestational ages reported in large international series [8, 9]. The open approach was chosen because laparoscopic TAC expertise and the requisite equipment were not yet established at our centre during the study period. All three patients presented already pregnant, precluding interval (nonpregnant) placement. The 12–13 week timing ensured that the cerclage was in place before the typical gestational window of cervical insufficiency. As demonstrated by the meta‐analysis of Hulshoff et al. [10], open laparotomy TAC during pregnancy yields comparable perinatal outcomes to the laparoscopic approach, with the trade‐off of longer hospital stay but shorter operative time.

### 3.1. Triplet Gestation (Case 2): An Outlier

Case 2, involving a triplet gestation delivered at 30 weeks 1 day, represents a clinical success in terms of neonatal survival for a high‐order multiple pregnancy with severe cervical insufficiency. However, this case is clearly identified as an outlier. The triplet pregnancy was complicated by preeclampsia and peripartum cardiomyopathy, both recognised risks of multiple gestation independent of cerclage status. Debiève et al. [11] previously reported favourable outcomes of TAC in twin pregnancies, but comparative evidence for triplets remains extremely limited. The contribution of the cerclage to the outcome in this case cannot be isolated from the confounding effects of the multiple gestation and its attendant obstetric complications. Therefore, this case should not be pooled with the singleton cases to support numeric claims of TAC efficacy.

### 3.2. Technical Considerations

From a technical perspective, all three procedures utilised the open laparotomy approach. Bladder dissection was performed in all cases by reflecting the vesicouterine peritoneum and mobilising the bladder inferiorly. The Mersilene tape was placed at the cervico‐isthmic junction—medial to the uterine vessels—and tied anteriorly at the level of the junction. The operative parameters (EBL: 50–200 mL; operative time: 75–140 min) are within the ranges reported for open procedures in international centres [10, 12].

The maternal complications observed in Case 2 (preeclampsia and peripartum cardiomyopathy) are recognised pathologies associated with high‐risk, multiple‐gestation pregnancies and are not known complications of cerclage surgery itself. Rather, the effectiveness of the TAC allowed the pregnancy to be maintained long enough for these obstetric risks to manifest.

This series serves as a proof of concept for implementing complex obstetric surgery within a Ghanaian specialist setting. It is critical to acknowledge that this success occurred within a private, well‐resourced hospital with specialised surgical expertise. These outcomes may not be generalisable to public or peripheral health facilities without similar surgical infrastructure. Translation of TAC to broader West African practice will require focused training, infrastructure investment and systemic support to ensure equitable access.

### 3.3. Rationale for Open Approach and Considerations for Future Practice

The open laparotomy approach was chosen for these three cases for several reasons. All patients presented already pregnant, precluding interval TAC placement. During the study period, laparoscopic TAC expertise and the required specialised instruments were not yet available at our centre; laparoscopic TAC is a highly complex procedure requiring advanced suturing skills that remain scarce in the West African subregion. The open approach, as validated by the meta‐analysis of Hulshoff et al. [10], produces comparable perinatal outcomes to the laparoscopic route. We acknowledge the work of Ades et al. [9], whose laparoscopic TAC series of 121 pregnancies achieved 98.5% perinatal survival, representing the modern benchmark for the procedure. As surgical capacity develops across the region, laparoscopic TAC may become an achievable goal. For the present context, however, the open approach represents the safest and most reliable option.

## 4. Limitations

This report has several limitations. As a retrospective case report from a single centre, the findings may not be generalisable. The small number of cases (*n* = 3) precludes statistical analysis and limits the strength of any comparative claims. Case 2 (triplet gestation) represents a major confounder, as multiple‐gestation‐related risks cannot be separated from cerclage effects. All procedures were performed at a private, well‐resourced specialist hospital. Adjunctive therapy with vaginal progesterone was administered to all three patients, and its independent contribution to the outcomes cannot be isolated. Larger, multicentre studies from across West Africa are needed to establish the true clinical utility of TAC in the region.

This report provides the first direct clinical evidence from West Africa documenting the feasibility of TAC for severe cervical insufficiency in a Ghanaian specialist setting. All three reported pregnancies resulted in live births, with outcomes comparable to international benchmarks. However, given the small sample and the confounders described, the findings should be interpreted as preliminary evidence of feasibility rather than proof of efficacy. This report supports the cautious integration of TAC into advanced obstetric practice in the region and highlights the need for future prospective multicentre studies.

**Nomenclature**
EBL: estimated blood loss
NICU: neonatal intensive care unit
PPCM: peripartum cardiomyopathy
TAC: transabdominal cerclage
TVC: transvaginal cerclage

## Author Contributions

R.M.K.D.: conceptualisation, write‐up, review and editing. R.M.K.D., E.A.A., K.A.N. and J.A‐K.: clinical diagnosis, surgical management and postoperative care of all three patients. E.A.A.: investigation, write‐up, review and editing. K.A.N.: write‐up and editing. J.A‐K.: review and editing. O.A. and A.P.V.: perioperative monitoring, follow‐up and editing. W.K.J.S‐A: editing. K.A.O‐A.: review and editing. A.A.O. and A.P.V.: perioperative monitoring and investigation.

## Funding

No funding was received for this manuscript.

## Disclosure

All authors approved the version to be published and agreed to be accountable for all aspects of the work.

## Ethics Statement

This study received ethical approval from the Committee on Human Research, Publications and Ethics of the Kwame Nkrumah University of Science and Technology, with reference number CHRPE/AP/1026/25.

## Consent

Written informed consent was obtained from the patients for publication of this case report and any accompanying images. A copy of the written consent is available for review by the editor‐in‐chief of this journal.

## Conflicts of Interest

The authors declare no conflicts of interest.

## Data Availability

Data sharing is not applicable to this article as no datasets were generated or analysed during the current study.
